# Comorbidity of physical illnesses and mental disorders in outpatients with tic disorders: a retrospective study using the outpatient case system

**DOI:** 10.3389/fneur.2024.1397766

**Published:** 2024-12-05

**Authors:** Liping Yu, Hui Xu, Zhongliang Jiang, Hanxue Yang, Yonghua Cui, Ying Li

**Affiliations:** ^1^Department of Psychiatry, Beijing Children’s Hospital, Capital Medical University, National Center for Children Healthy, Beijing, China; ^2^Big Data Center, Beijing Children's Hospital, Capital Medical University, National Center for Children's Health, Beijing, China; ^3^School of Psychology, Beijing Language and Culture University, Beijing, China; ^4^Department of Psychosomatic Medicine, Beijing Children’s Hospital, Capital Medical University, National Center for Children Healthy, Beijing, China

**Keywords:** tic, Tourette syndrome, comorbid, physical disease, outpatient, retrospective analysis

## Abstract

**Background:**

Tic disorder, a chronic neurodevelopmental disorder that typically onsets during childhood, is characterized by sudden, involuntary, rapid, and non-rhythmic motor and vocal tics. Individuals with tic disorders often experience physical health issues. The purpose of our retrospective analysis was to elucidate the common comorbid physical diseases and mental disorders and their characteristics of outpatient children with tic disorders in a large public children’s hospital in China over the past 5 years.

**Methods:**

Participants were outpatients with tic disorders who visited Beijing Children’s Hospital, from January 1, 2018 to December 31, 2022. After the inclusion screening, a total of 523,462 patient visits were included in the analysis. Based on the International Classification of Diseases, 10th Revision (ICD-10) diagnostic system, we employed descriptive statistical analysis to examine the frequently co-occurring somatic diseases in patients with tic disorders, as well as the influence of variables such as age and seasonal variation on these comorbidities.

**Results:**

The top five diseases of total outpatient visits were as follows: Respiratory diseases, Mental and behavioral disorders, Diseases of the eye and adnexal, Digestive disorders, Diseases of the skin and subcutaneous tissue. Among the top five comorbid disease system, the most commonly third- level classification of diseases were upper respiratory tract infections, attention deficit and hyperactivity disorder, conjunctivitis, dyspepsia, dermatitis. Respiratory system diseases experienced a peak in April, while the other four types of diseases reached their peak in August. Additionally, each disease system showed the lowest number of patient visits in February. Additional to the mental and behavioral disorders, the other four disease systems would experience a peak in medical visits between the ages of 4 and 6.

**Conclusion:**

Our study highlighted the most common physical diseases and mental disorders in tic disorders, namely the respiratory diseases, specifically upper respiratory tract infections, and mental and behavioral disorders, with ADHD being the most common co-occurring condition.

## 1 Introduction

Tic disorder, a chronic neurodevelopmental disorder that typically onsets during childhood (between the ages of 4–7), is characterized by sudden, involuntary, rapid, and non-rhythmic motor and vocal tics (1–3). The prevalence of common childhood psychiatric disorders, such as tic disorder, attention deficit hyperactivity disorder (ADHD), and autism spectrum disorder (ASD), has been found to be higher in boys than in girls on a global scale (4–6). According to a 2018 epidemiological survey of children aged 6–16 in China, the prevalence of tic disorder was 2.5%, whose incidence significantly declined among adolescents aged 12 and older compared to the group aged 6–11 years (5), consistent with previous research (3, 7–9).

Tic disorder has been found to be highly comorbid with psychiatry disorders such as ADHD and OCD. However, exploration on comorbidity of tic disorder with physical diseases are rare (10). Recent studies have further confirmed the increasingly important role of autoimmune diseases in the onset of tic disorder (11–13). Previous studies have found a correlation between tics and autoimmune encephalitis, with tics being the most common movement disorder in the later stages of encephalitis, and in the aggressive treatment of encephalitis, the patient’s tics improved (14, 15). This may be related to the presence of anti-basal ganglia antibodies (ABGA) in the basal ganglia of patients with autoimmune encephalitis, which contribute to the development of ABGA tics (16). Reviews and studies on multigenerational family clusters suggested a familial aggregation of autoimmune diseases in relation to tic disorders (12, 17). The clinical concept of pediatric acute-onset neuropsychiatric syndrome (PANS) includes an acute onset of obsessive-compulsive behavior or food restriction with at least two severe acute onset neuropsychiatric symptoms (e.g., cognitive, and behavioral symptoms) (18). Inflammation and immune response mediated by Group A Streptococcus (GAS) infection play a predominant role in the development of this disorder (19). Previous studies have also found that patients with tic disorders with PANS have a different clinical presentation and comorbidities than tic patients without PANS, and have identified tic patients with PANS who have reduced levels of M antibodies and elevated CD8+ lymphocytes, findings that may predict the development of autoimmune neuropsychiatric disorders in patient populations (20). Pediatric autoimmune neuropsychiatric disorders (PANDAS) are considered a subset of PANS (18). Previous studies have found a correlation between PANDAS associated with GAS infection and tics, and have noted a clear temporal correlation between Streptococcal infection and the onset or exacerbation of tic symptoms in prepubertal children (21–23). Concurrent treatment of infection, inflammation, and psychiatric and behavioral symptoms in patients with comorbid PANS or PANDS has been found to be helpful in improving patients’ quality of life and social functioning (20).

A 40-year long-term cohort study revealed significant associations between tic disorder and metabolic and cardiovascular risks (24). Another review suggested that the ocular symptoms of benign essential blepharospasm and tic disorder exhibit similar manifestations (25), potentially indicating a common pathophysiological mechanism between ocular tics and blepharospasm (26). The latest research has found that compared to the normal control group, children with tic disorder exhibited lower meibomian gland length and area in their eyes (27). According to Baizabal-Carvallo et al. (28), oral tics were relatively less frequent, but in some cases, they may lead to severe self-injurious damage to oral tissues (29, 30). Budman (31) reported that oral herpesvirus infection potentially exacerbated the psychological and behavioral symptoms of tic disorders. Recent study has indicated an increase in *Helicobacter pylori* and Bacteroides in the gastrointestinal tract of tic disorder with coprolalia (32). However, current research on comorbid physical diseases in tic disorders is limited, necessitating further investigation. This area of study holds significant clinical value for both the prevention and treatment of tic disorders.

Furthermore, the latest study suggested a possible association between tic disorder and allergic diseases (33–35). Previous studies have also confirmed a positive correlation between tic disorder and asthma, allergic rhinitis, allergic conjunctivitis and eczema with the reason that tic disorders may have the same genetic susceptibility as allergic diseases. Among these, the diagnosis rate of allergic rhinitis was notably higher (36–38). Nevertheless, it is difficult to accurately determine which specific allergic disease is more likely to be accompanied by specific tic disorders (33). A study on serum immunoglobulin E (IgE) levels in children aged 6–9 with tic disorder showed higher IgE levels compared to the healthy control group (39). During the coronavirus disease 2019 (COVID-19) pandemic, it was observed that tic symptoms could worsen during respiratory virus infections (40, 41). Hoekstra (42) found that, compared to the adult Tourette’s disorder group, symptoms in the pediatric group worsen around 4 weeks after contracting a common cold. Therefore, investigating the physical diseases associated with tic disorders is of great significance for the management based on tic disorders and can effectively reduce the fluctuation of tic symptoms.

The purpose of our retrospective analysis is to elucidate the characteristics of children with tic disorder and the comorbidity of physical diseases in tic disorder patients. This analysis would include changes in annual outpatient volumes, comorbidity patterns with which physical diseases, the most common physical diseases, comorbidity rates of psychiatric disorders, age of onset, gender differences, and seasonal variations in outpatient visits. The findings might provide a better basis for preventing fluctuations in tic disorder symptoms and the comorbidity of physical diseases in the future.

## 2 Materials and methods

### 2.1 Participants

The included sample consisted of all children with tic disorders who visited the outpatient department of Beijing Children’s Hospital of Capital Medical University (National Center for Children’s Health, China), from January 1, 2018 to December 31, 2022. Over the past 5 years, patients with tic disorders have sought medical attention at this hospital at least once due to physical illnesses. Patients with tic disorders and common physical illnesses and mental disorders met the diagnostic criteria of the International Statistical Classification of Diseases, 10th revision (ICD-10). We carefully crafted the inclusion and exclusion criteria to accurately delineate the participant cohort for our study.

The ICD-10 diagnostic system is a structured, multilevel classification that organizes diseases and health conditions into a comprehensive hierarchy for global health statistics and information. At the broadest level are the chapters, which categorize conditions into groups influenced by the affected body system or the nature of the disease. Within these chapters, conditions with common characteristics are further grouped into blocks. These blocks are then subdivided into categories, typically defined by the disease’s etiology and the body system it impacts. To achieve greater precision, categories can be divided into subcategories, detailing the specifics of the condition. Moreover, for enhanced detail, extension codes are used to encapsulate particulars such as the cause of an injury or how a disease presents itself. In this study, we have primarily utilized the first three levels of the ICD-10 diagnostic hierarchy, which includes chapters (first-level classification), blocks (second-level classification), and categories (third-level classification), to classify disease diagnoses.

**Inclusion criteria:**

1. Children aged below 18 years;
2. Diagnoses of physical and mental illnesses falling under the second-level classification of ICD-10, comply with the diagnostic criteria in ICD-10;
3. Children with tic disorders who have experienced at least one type of physical illness in the past 5 years.

**Exclusion criteria:**

1. Comorbid mental disorders not related to third-level classification;
2. Comorbid physical illnesses not related to third-level classification.

**Figure 1:**
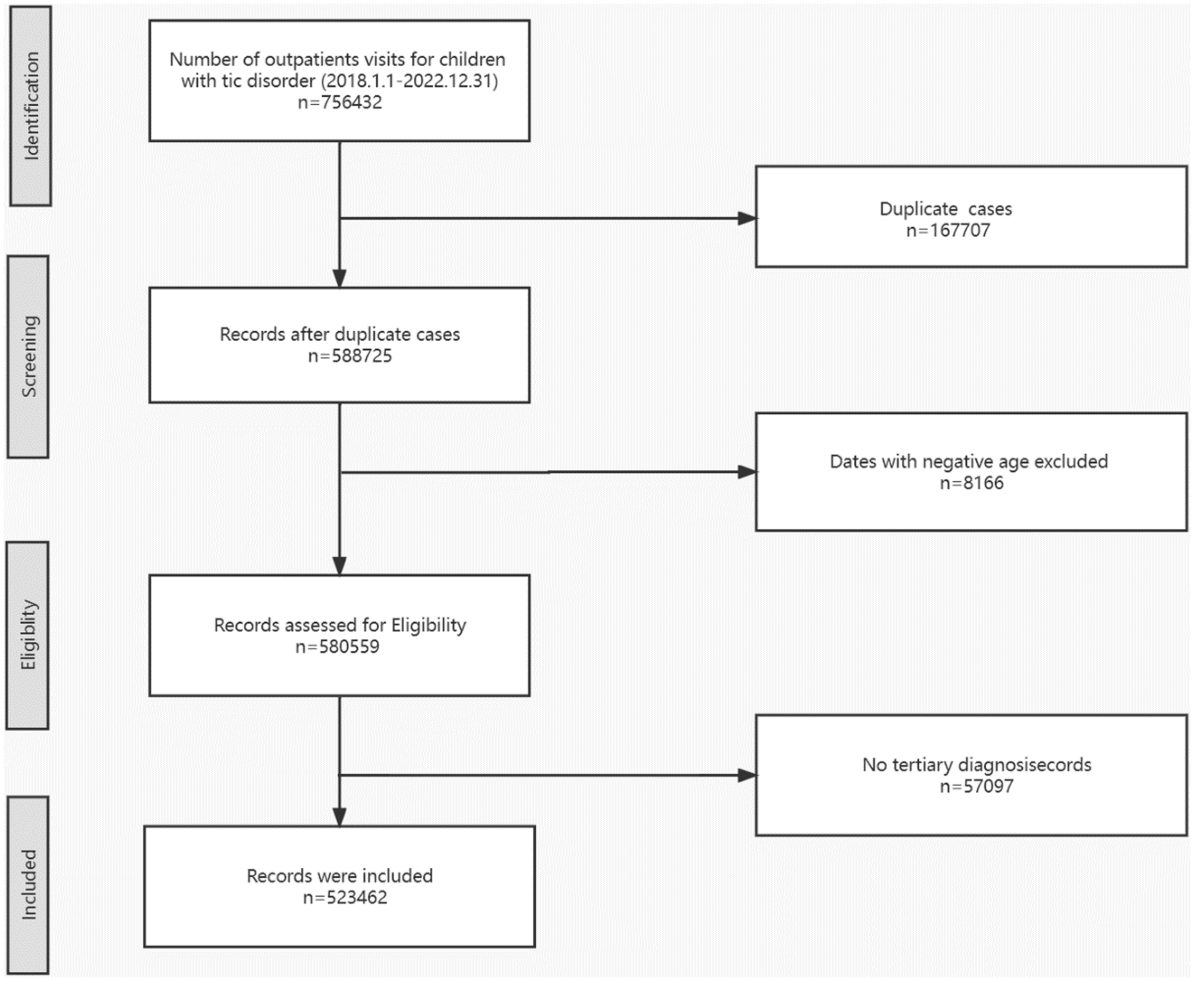
Flow diagram for the identification of cases in the study.

Following the specified Inclusion and Exclusion criteria, our study analyzed a cohort consisting of 41,788 patients (31,808 males and 9,980 females), with a total of 523,462 medical records of patients diagnosed with tic disorders. Our study focuses on the possible co-occurrence of other diseases in different individuals at different times, so we conducted a longitudinal analysis. The same patient with tic disorders who comes to the clinic may have a different comorbidity at each visit. To better capture this information, we included 523,462 patient visits for the final analysis. For more details, please see Figure 1.

### 2.2 Data collection

We collected data on the number of outpatient visits for tic disorders who concurrently had common physical diseases and mental disorders over a five-year period. Specifically, this included (1) the annual number of outpatient visits per patient, (2) the total number of outpatient visits over the 5 years for each patient; (3) the total number of outpatient visits per year; (4) the total number of outpatient visits over the 5 years for each physical disease; (5) the total number of outpatient visits per month for each physical disease; (6) the total number of male and female patients for each physical disease; (7) the total number of outpatient visits for each age group for each physical disease; (8) the total number of outpatient visits over the 5 years for patients diagnosed with the second-level classification of the ICD-10 in each physical disease; and (9) the total number of outpatient visits over the 5 years for patients diagnosed with the third-level classification of the ICD-10 in each physical disease. For patients in each physical disease where the second-level classification and third-level classification were involved, the data from the top five categories based on the total number of outpatient visits were selected for analysis.

### 2.3 Data analysis

Continuous variables that conformed to normal distribution were described as mean ± standard deviations (SDs). Due to the skewed distribution of the hospitalization duration, its median with interquartile ranges (IQRs) was also used and presented. All categorical variables in the study were presented as counts.

To better show the distribution of the diseases, the top five first-level classification of the diseases in the ICD-10 were described in total, as well as by year, mouth, sex and age, respectively. Then the top five second-level classification and third-level classification of the diseases under the top five first classification in the ICD-10 were also presented. All statistical analyses in this study were conducted with John’s Macintosh Product (JMP) Pro 17.0.

## 3 Results

### 3.1 The annual outpatient visits and total outpatient visits over these 5 years

From January 1, 2018 to December 31, 2022, the five-year annual average visitation data indicated that the annual average number of visits in 2018, 2019, and 2021 were quite similar, all of which were higher than those in 2020 and 2022. These visits included a total of 381,501 (72.88%) males and 141,961 (27.12%) females. The mean age of the participants was 6.41 ± 3.17. See Table 1.

**Table 1:** General information and age distribution of samples.

Sample	Visit	Individuals	Age	SD	95%CI
Total	837,801	52,953	7.07	3.207	(7.06, 7.08)
Male	620,626	40,416	7.14	3.151	(7.13, 7.15)
Female	217,175	12,537	6.86	3.354	(6.85, 6.88)
Child	655,612	43,119	5.79	2.162	(5.78, 5.79)
Adolescent	182,189	17,012	11.69	1.783	(11.68, 11.70)

SD, standard deviation; CI, confidence interval.

The annual average number of visits in 2020 was the lowest over these 5 years (2.0829 ± 5.4782). Please refer to Table 2 for details. Meanwhile, the total outpatient visits also underwent corresponding changes.

**Table 2:** Average outpatient visits in different years.

	2018	2019	2020	2021	2022	Total
Mean	3.14162	3.343496	2.082919	3.029745	2.490595	14.08838
SD	6.789098	7.01765	5.478238	7.123194	6.79045	23.26383
Quartile 25	1	1	1	1	1	6
Median	1	1	1	1	1	2
Quartile 75	3	4	2	3	2	16

SD, standard deviation.

### 3.2 The number of visits varies with the month

**Figure 2:**
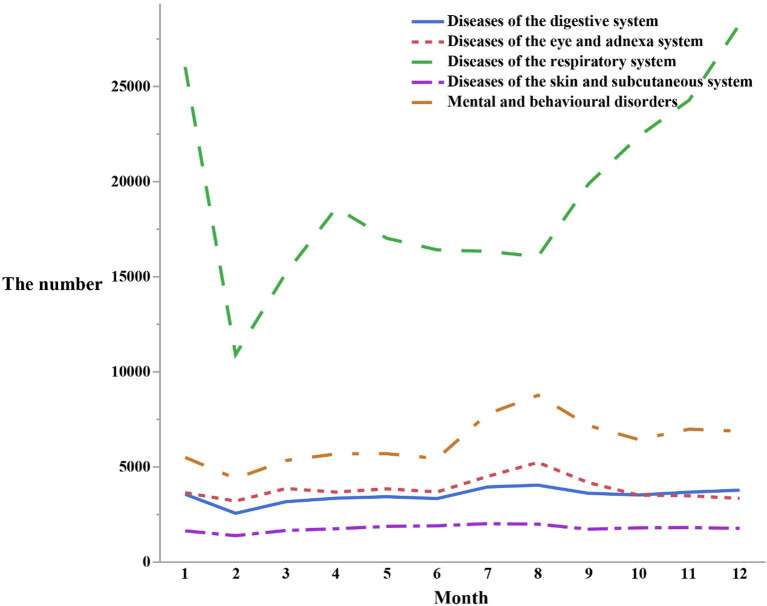
The number of outpatients visits in different months by the top five first classification of the diseases in the ICD-10.

In February, relatively lower peaks were found in visits for respiratory diseases (*n* = 10,906), mental and behavioral disorders (*n* = 4,398), eye and adnexal disorders (*n* = 3,211), digestive disorders (*n* = 2,566), and skin and subcutaneous tissue disorders (*n* = 1,390). However, in August, a significant increase in visits for mental and behavioral disorders (*n* = 8,773), eye and adnexal disorders (*n* = 5,240), digestive disorders (*n* = 4,046), and skin and subcutaneous tissue disorders (*n* = 1,995) was noted. Respiratory disorders also experienced a peak in August (*n* = 16,065). These findings indicated that respiratory disorders exhibited more pronounced fluctuations compared to the other four disorders, which demonstrated a relatively smoother pattern of fluctuation (Figure 2).

### 3.3 The number of visits varies with age

**Figure 3:**
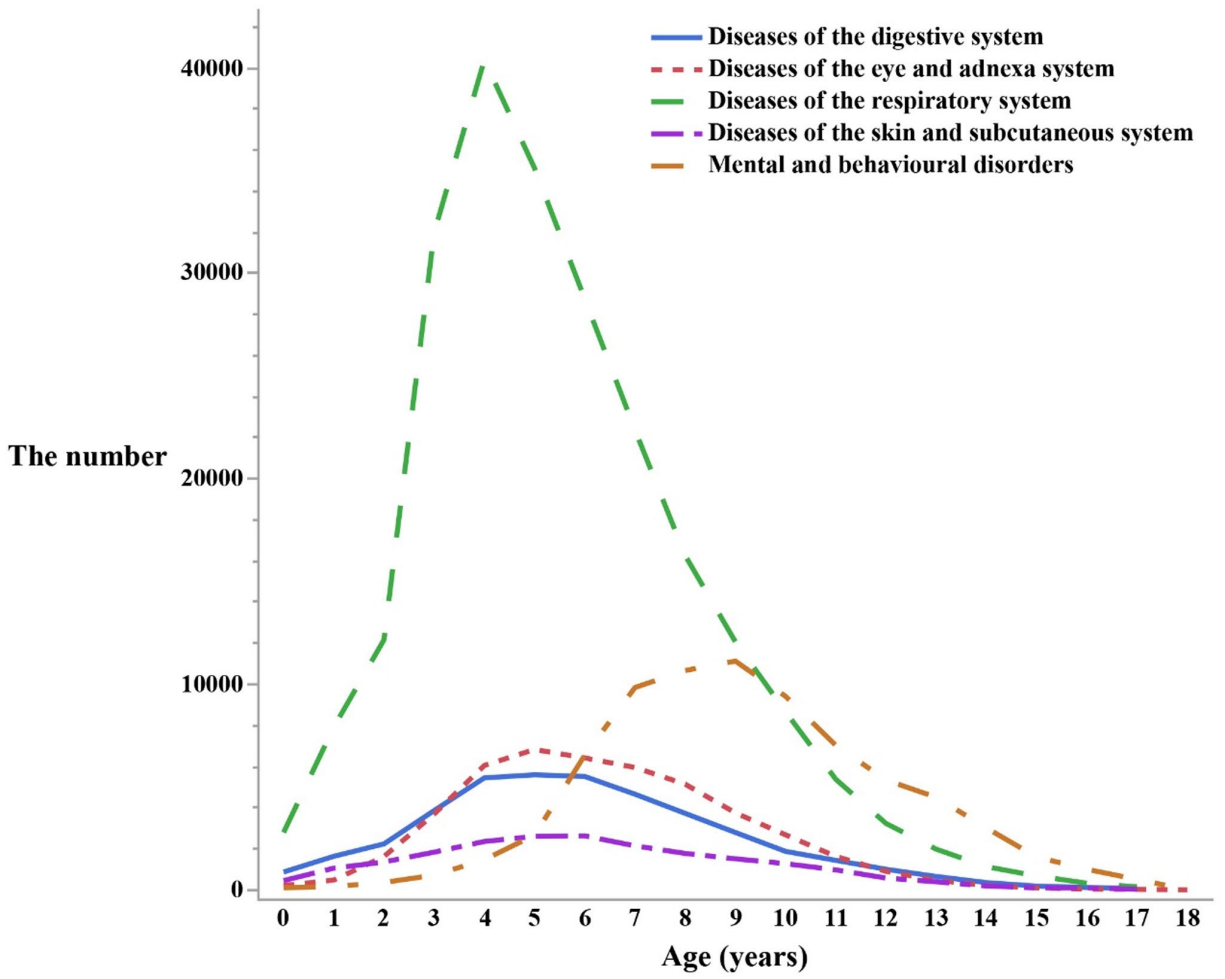
The number of outpatients visits in different ages by the top five first classification of the diseases in the ICD-10.

Respiratory disorders reached their peak number of visits around the age of 4, while mental and behavioral disorders exhibited the highest visitation rates at approximately 9 years of age. Eye and appendage disorders showed a peak period of visits at around 5 years old, and digestive disorders tended to have the highest number of visits between the ages of 4 and 6. Similarly, skin and subcutaneous tissue disorders peaked in terms of visits at approximately 6 years old. Prior to reaching the peak period, visits tended to increase as individuals age, and after the peak period, they gradually declined. It has been recognized that a gradual remission would commonly begin after puberty, typically around the age of 12. (For a visual representation of these trends, please refer to Figure 3).

### 3.4 The top five diseases for annual outpatient visits and total outpatient visits over these 5 years

**Figure 4:**
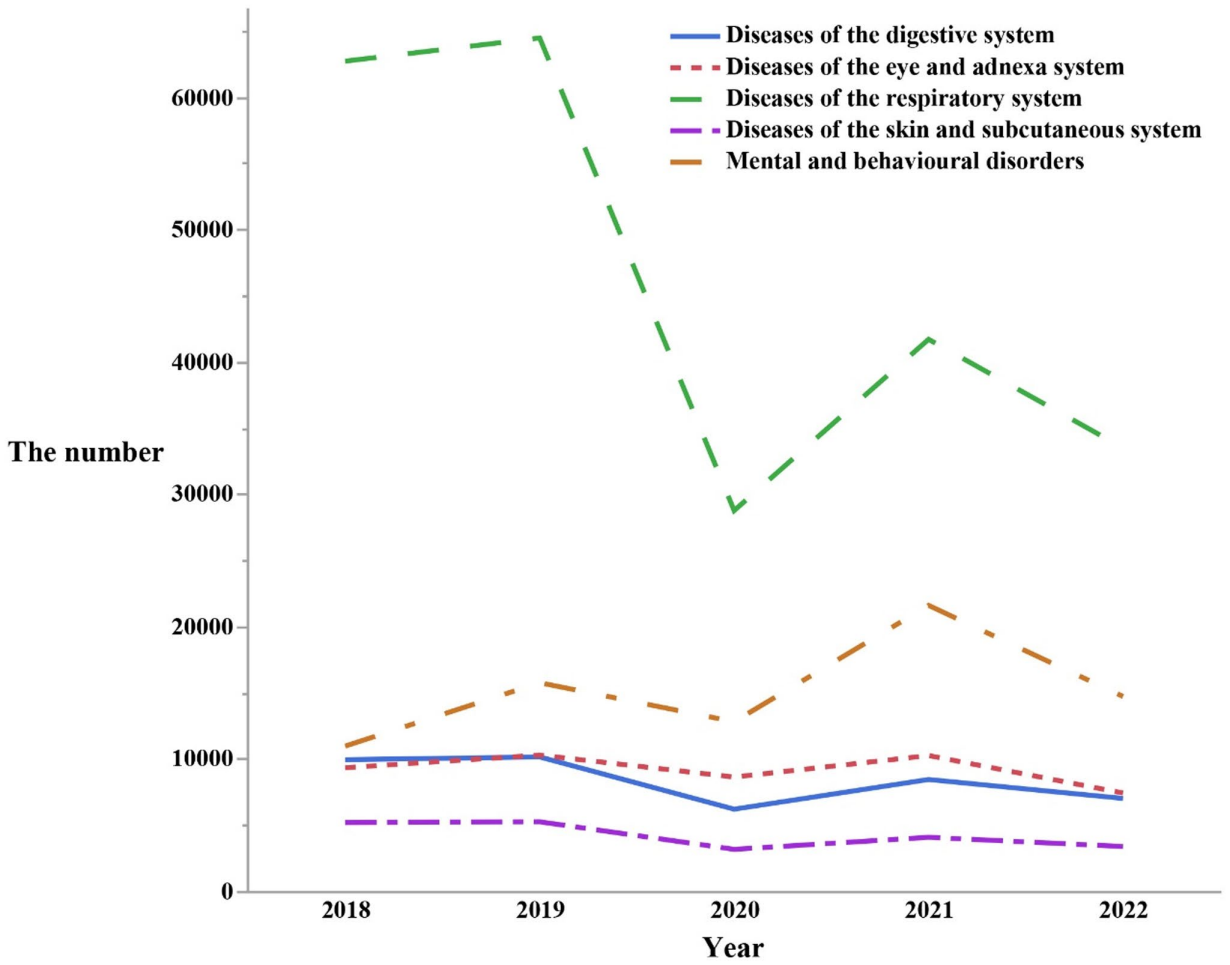
The number of outpatients visits from 2018 to 2022 by the top five first classification of the diseases in the ICD-10.

Diseases ranked in descending order of quantity were as followed: Respiratory diseases (*n* = 231,432), Mental and behavioral disorders (*n* = 76,157), Diseases of the eye and adnexal (*n* = 46,240), Digestive disorders (*n* = 42,063), Diseases of the skin and subcutaneous tissue (*n* = 21,385) (Figure 4). Characteristics of changes were also noted: The total number of outpatient visits for these five disease systems was the highest on average in the year 2019. Subsequently, in 2021, another peak in outpatient visits presented, but was lower than that of 2019. In the remaining 3 years, each disease system exhibited its own distinctive patterns of changes in the total number of outpatient visits. Respiratory diseases showed a slight increase in total outpatient visits in the years 2018, 2020, and 2022. The outpatient visits patterns for mental and behavioral disorders, diseases of the eye and adnexal, digestive diseases, and diseases of the skin and subcutaneous tissue all indicated that 2020 had the lowest total number of visits. In 2022, the total number of visits approached the levels of 2020 but remained lower than those in 2018. Interestingly, diseases of the skin and subcutaneous tissue exhibited a much smaller fluctuation in the total number of outpatient visits over the past 5 years compared to the other four disease systems.

### 3.5 The top five third-level classification of diseases in terms of their respective total number of visits

Among respiratory disorders, mental and behavioral disorders, diseases of the eye and adnexal, digestive disorders, diseases of the skin and subcutaneous tissue, the most common third-level classification of the diseases were, respectively, upper respiratory tract infections, ADHD, conjunctivitis, dyspepsia, and dermatitis (Table 3).

**Table 3:** The top five tertiary categorized diseases in terms of their respective total number of visits.

Disease systems	Third-level classification of diseases				
Respiratory disorders	Upper respiratory tract infections *n* = 74,593	Colds *n* = 43,071	Rhinitis *n* = 40,038	Pharyngitis *n* = 17,740	Coughs *n* = 9,782
Mental and Behavioral Disorders	Attention Deficit and Hyperactivity Disorder *n* = 47,090	Psychotic Disorder *n* = 10,156	Childhood Mood Disorder *n* = 4,155	Depressive State *n* = 2,959	Childhood Autism *n* = 2,498
Diseases of the eye and adnexal	Conjunctivitis *n* = 18,710	Refractive error *n* = 15,690	Dry eye *n* = 6,892	Keratitis *n* = 1,672	Chalazion *n* = 1,118
Digestive disorders	Dyspepsia *n* = 9,521	Gastrointestinal disorders *n* = 7,512	Diarrhea disorders *n* = 3,320	Gastroenteritis *n* = 3,064	Constipation *n* = 2,688
Diseases of the skin and subcutaneous tissue	Dermatitis *n* = 6,193	Eczema *n* = 4,762	Urticaria *n* = 3,273	Rash *n* = 1,802	Pompholyx *n* = 731

### 3.6 For each disease, the top five second-level classification of disease are listed below

**Figure 5:**
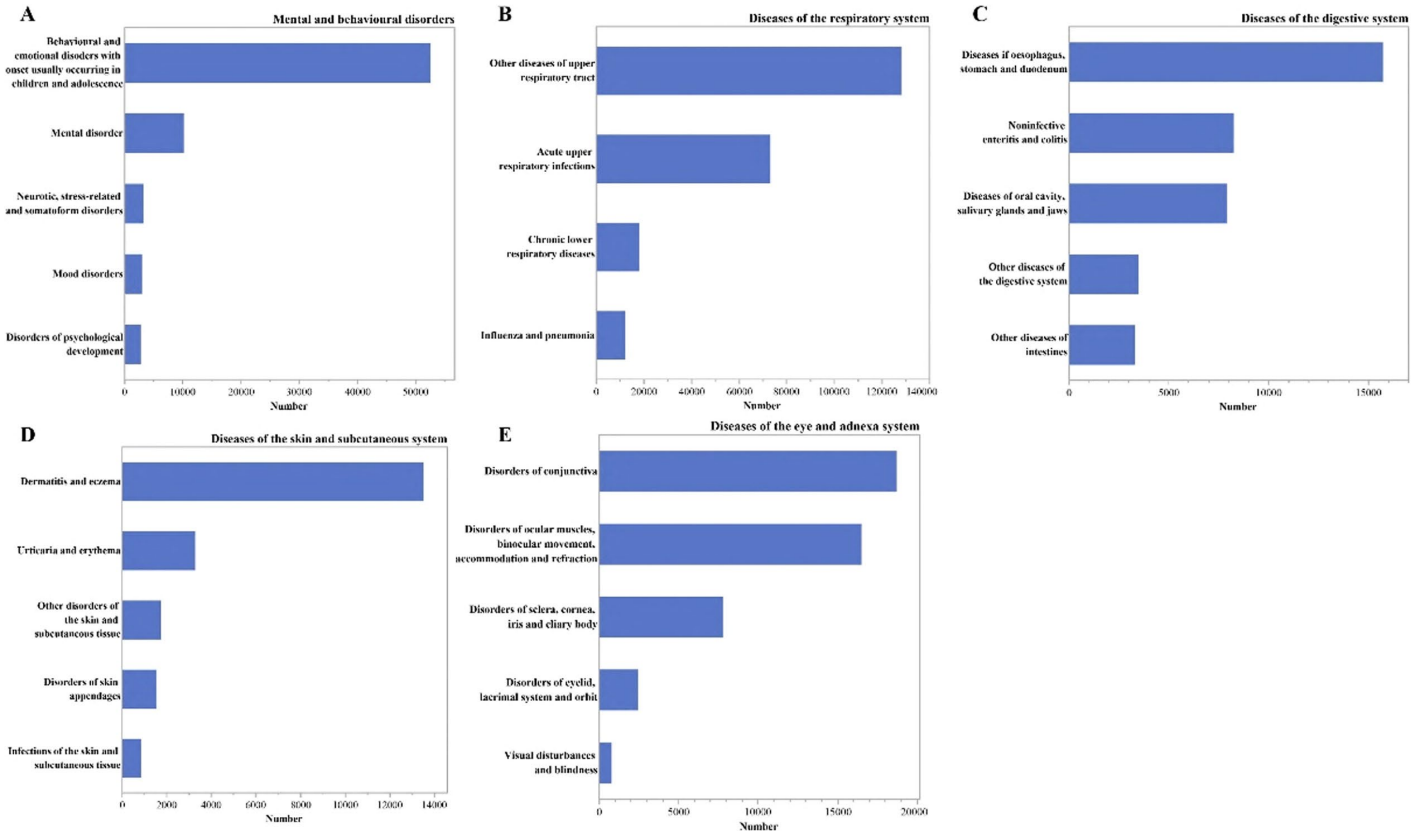
The number of outpatients visits by the secondary classification of the diseases in the ICD-10. **(A)** Under the mental and behavioral disorders; **(B)** under the diseases of the respiratory symptom; **(C)** under the diseases of the digestive symptom; **(D)** under the diseases of the skin and subcutaneous tissue symptom; **(E)** under the diseases of the eye and adnexal symptom.

The most common second-level classification of diseases for respiratory disorders, mental and behavioral disorders, diseases of the eye and adnexal, digestive disorders, and diseases of the skin and subcutaneous tissue were as followed: respiratory diseases comprised of other upper respiratory diseases (*n* = 128,174), mental and behavioral disorders commonly observed in children and adolescents (*n* = 52,578), diseases of the conjunctiva (*n* = 18,710), esophageal, gastric, and duodenal disorders (*n* = 15,713), dermatitis and eczema (*n* = 13,529). For more details, refer to Figure 5.

## 4 Discussion

Our retrospective study aimed to investigate the characteristics of coexisting somatic and mental disorders in children with tic disorders attending public children’s hospitals. Most importantly, common physical diseases in tic disorder included respiratory system disorders, eye and adnexal diseases, digestive disorders, and skin and subcutaneous tissue diseases. Among these, the most common comorbid diseases associated with tic disorders were upper respiratory tract infections, conjunctivitis, indigestion, and dermatitis.

### 4.1 Characteristics of comorbidity in the second-level classification and third-level classification

Our study found that respiratory disorders were the most commonly comorbid somatic diseases with tic disorders, with other upper respiratory tract diseases being the most common in the second-level classification. Furthermore, among the third-level classification, upper respiratory tract infections were the most common. This suggested a possible association between tic disorders and respiratory tract infections, in accordance with previous studies (43, 44). Previous research has shown that respiratory tract infections triggered by streptococcal infections could lead to autoimmune psychiatric disorders, leading to exacerbation of tic symptoms (45–47). Several studies have already shown that the COVID pandemic has a significant impact on the mental health of young people with TS, exacerbating both tics and emotional and behavioral symptoms (48, 49). An increase in tic symptoms has been reported in some children and adolescents with tic disorders since the onset of the COVID-19 pandemic (49, 50). In addition, another recent study aimed to explore the long-term effects of SARS-CoV-2 infection in children and adolescents with tic disorders reported that patients infected with severe acute respiratory syndrome coronavirus 2 (SARS-CoV-2) had more pronounced tics and were more likely to have comorbidities than uninfected patients, suggesting that infection may contribute to the increase in tics and associated comorbidities in patients with tics (51). Some studies have indicated a significant increase in the incidence of movement disorders during the COVID-19 pandemic, such as functional tic disorders (52). Functional tics belong to functional abnormal movement, which phenomenologically shows common features with tic disorders (53). The latest study found an increase in the number of people suffering from functional tics during the COVID-19 pandemic (53, 54). These patient populations are predominantly prevalent in adolescent females and young adult females, mostly suffering from anxiety disorders as well as having been exposed to social media content related to tics, but mostly not benefiting from tic suppression medication (54). A prospective study of functional tics noted that adolescents with functional tics had a better prognosis than adults (55). However, other studies reported no significant association between streptococcal infections and exacerbation of tic symptoms (56).

The most prevalent second-level classification of eye and adnexal disorders associated with tic disorder was conjunctival disease, which was further categorized into tertiary classifications, with conjunctivitis as the most common subtype. Several children attending the tic clinic have documented visits to ophthalmologists, who were also diagnosed as “allergic conjunctivitis.” Previous meta-analyses have indicated a potential link between the development of tic disorder and allergic diseases, with tic disorder showing a positive correlation with allergic asthma, allergic rhinitis, and allergic conjunctivitis (33, 57). A recent study focusing on the relationship between allergic conjunctivitis and tic disorders in 4–10-year-olds in southwestern China found that transient tic disorders exhibited a higher frequency of allergic conjunctivitis, suggesting a potential association between allergic conjunctivitis, dry eye, and transient tic disorders in children (58). Furthermore, a previous study conducted in Taiwan, which involved 99% coverage of the population, revealed that patients with tic disorders had a higher likelihood of experiencing allergic disorders (59).

The most prevalent second-level classification of diseases that often co-occur with tic disorders and involve the digestive system were esophageal, gastric, and duodenal diseases. When further divided into tertiary categories, the most common disorder was dyspepsia. Previous research has indicated that individuals diagnosed with “aerophagy” experience symptoms like abdominal pain, bloating, and vomiting, which were initially treated symptomatically as dyspepsia (60). However, further investigation revealed that frequent swallowing of air due to tic disorders was the underlying cause of their abdominal discomfort (61, 62). It has also been observed that the incidence of tic disorders combined with aerophagy was higher in China compared to that of aerophagy alone (63).

Regarding skin and subcutaneous tissue disorders that frequently co-occur with tic disorders, dermatitis and eczema were the most common second-level classification, which were further divided into third-level classification, with dermatitis being the prevailing co-occurring disorder. Previous studies have found that thimerosal-containing vaccines increased the chances of children developing contact dermatitis and an elevated risk of developing tic disorders (64). A previous case–control study demonstrated a significant correlation between atopic dermatitis and tic disorders (57), suggesting that histaminergic neurotransmission may play a crucial role in the pathogenesis of tic disorders (65, 66).

### 4.2 Characteristics of comorbidity other neuropsychiatric disorders

Our study revealed a relatively high likelihood of comorbidity of psychiatric and behavioral disorders in individuals with tic disorder. Psychiatric and behavioral disorders were the second most common disease system across all tic disorders, which aligns with previous research indicating that a majority of children with tic disorders will have at least one co-occurring psychiatric disorder (9, 10, 67). Our study specifically identified ADHD, childhood mood disorders, depressive states, and childhood autism as common psychiatric disorders that often co-occur with tic disorder. These findings were consistent with previous studies reporting associations between tic disorders and ADHD, dysphoria, anxiety, and ASD (10, 68, 69).

Prior research has shown that the most frequent co-morbid psychiatric disorders in chronic tic disorders were ADHD and OCD (67, 70). Regarding the comorbidity of mental disorders with tic disorders, the most common second-level classification of diseases were behavioral and mental disorders that usually occur in children and adolescents, corresponding to the third-level classification, ADHD ranked as the top co-morbid psychiatric disorder, consistent with previous studies (9, 68). A previous study found an association between emotional dysregulation and severe tic symptoms (71). A recent study also reported that patients with tic disorder were more likely to be depressed than those without the disorder and that there was an association between depression and the severity of tic disorder in children and adolescents (72). This is also in line with previous research findings (73).

Previous studies have found that epilepsy affects autonomic activity, and changes in this area have the potential to influence changes in tic symptoms (74). Stress and anxiety predispose to epilepsy, which is similar to tics (71, 75, 76). Some studies have found that the pathogenesis of tics in patients involves immune mechanisms (77), and there is an association between autoimmune encephalitis associated with streptococcal infection and tic disorders (14, 78). In addition, studies have found that tic disorders may have abnormalities in gait and postural control (78–80). There’s been a case report of severe tics leading to cervical spondylosis (81).

### 4.3 Characteristics of season and year of consultation

Previous research has indicated that the incidence of tic disorders may be higher during the winter months compared to the spring, with a higher prevalence observed between November and February (2, 82). However, our study found a higher consultation rate during July and August, although this does not necessarily indicate a higher incidence during these months. This observation may be attributed to the specific national situation of our country. July and August marked as summer vacation period for children and adolescents in China, justly, the previous study has shown there is a regular holiday pattern in the months of visits for some pediatric diseases (83). During this time, children are not attending school, and families do not have to worry about disrupting their children’s classes or taking time off from work, making it more convenient for them to bring their children to the hospital for consultations.

In our analysis of the past 5 years, we observed a relatively stable number of visits for tic disorders in the years 2018, 2019, and 2021, with minimal differences. However, there was a significant decrease in the number of visits in 2020 and 2022, particularly in 2020, which was most noticeable. This trend did not align with the prevalence of tic disorders in the past (5, 84, 85). It is important to note that the decrease in the number of visits during these years does not imply that the prevalence of patients with tic disorders decreased. Rather, it may be related to the prevention and control measures implemented during the COVID-19 pandemic in China (86). Factors such as lockdown measures, strict controls, and quarantine protocols may have prevented patients from seeking timely medical attention at hospitals.

## 5 Limitations

This study has three limitations. Firstly, our study was limited to a single center, the National Children’s Medical Center at Beijing Children’s Hospital of Capital Medical University, leading to incomplete sample coverage and potentially limiting the generalizability of our findings. Secondly, the data used in our study were derived solely from outpatient clinics, and thus, the profiles of inpatient hospitalizations were not included, and some patients did not visit the hospital, potentially resulting in incomplete coverage of diseases. Thirdly, some patients with tic disorder were diagnosed with non-psychiatric diseases during visits to non-psychiatric departments. For example, tic disorder symptoms such as blinking may be misdiagnosed as conjunctivitis, or involuntary movements may be erroneously used as diagnostic descriptions. These factors introduced the possibility of errors in disease classification during data collection.

## 6 Conclusion

Our study highlighted the most common physical diseases and mental disorders in tic disorders were the respiratory diseases, specifically upper respiratory tract infections, and mental and behavioral disorders, with ADHD being the most common co-occurring condition. The observed patterns in the seasons and years of visits for these comorbidities reflected our specific national circumstances, including the implementation of epidemic prevention and control measures, as well as the concentration of visits during summer and winter vacations.

## Data Availability

The datasets presented in this article are not readily available because due to confidentiality agreements with the participants, we are unable to publicly disclose the data. In case of special circumstances, please contact the corresponding author to request access to the data. Requests to access the datasets should be directed to dongyuzui1@163.com.
